# Real time monitoring of water level and temperature in storage fuel pools through optical fibre sensors

**DOI:** 10.1038/s41598-017-08853-7

**Published:** 2017-08-18

**Authors:** S. Rizzolo, J. Périsse, A. Boukenter, Y. Ouerdane, E. Marin, J-R. Macé, M. Cannas, S. Girard

**Affiliations:** 1 Laboratoire Hubert Curien, UMR-CNRS 5516, 42000 Saint-Etienne, France; 2 Areva NP, 69006 Lyon, France; 3 Dipartimento di Fisica e Chimica, Università di Palermo, 90128 Palermo, Italy; 4 Areva, Paris-La Défense, France

## Abstract

We present an innovative architecture of a Rayleigh-based optical fibre sensor for the monitoring of water level and temperature inside storage nuclear fuel pools. This sensor, able to withstand the harsh constraints encountered under accidental conditions such as those pointed-out during the Fukushima-Daiichi event (temperature up to 100 °C and radiation dose level up to ~20 kGy), exploits the Optical Frequency Domain Reflectometry technique to remotely monitor a radiation resistant silica-based optical fibre i.e. its sensing probe. We validate the efficiency and the robustness of water level measurements, which are extrapolated from the temperature profile along the fibre length, in a dedicated test bench allowing the simulation of the environmental operating and accidental conditions. The conceived prototype ensures an easy, practical and no invasive integration into existing nuclear facilities. The obtained results represent a significant breakthrough and comfort the ability of the developed system to overcome both operating and accidental constraints providing the distributed profiles of the water level (0–to–5 m) and temperature (20–to–100 °C) with a resolution that in accidental condition is better than 3 cm and of ~0.5 °C respectively. These new sensors will be able, as safeguards, to contribute and reinforce the safety in existing and future nuclear power plants.

## Introduction

Fukushima-Daiichi event on March 11^th^, 2011, signed a turning point in nuclear industry by highlighting several weaknesses in the control of the critical systems ensuring the safety in nuclear power plant (NPP), in particular when they are exposed to harsh constraints associated to extreme and accidental conditions. The main issue in stake for the nuclear industry is to enhance the security and improve the durability of the existing and future NPPs. During abnormal transients or accident conditions in nuclear reactors, key thermodynamic parameters (e.g., temperature, pressure, and water level in the reactor vessel; temperature, pressure, and radiation level in the containment; water level and temperature in spent (or storage) fuel pools, SFPs) must be known to facilitate appropriate operator actions. Indeed, the reliability of information gained from the instruments is a key to decision making and action taking by operators. The Fukushima Daiichi accident demonstrates the need to further harden essential reactor, containment, and SFP monitoring instrumentation to better withstand severe-accident conditions. The U.S. nuclear industry and the USNRC have already recognized the need for enhanced reactor and containment monitoring instrumentation, in particular with respect to monitoring SPF water levels^1^. Furthermore, what happened in Fukushima-Daiichi NPP pointed out new needs, both in terms of parameters to be monitored and of new technologies able to survive to the increase of temperature and radiation dose levels during such events to maintain the safety requirements^1, 2^. The post-Fukushima climate stimulates the research for the development of qualified radiation resistant sensors based on optical fibres. Indeed, since their first appearance during late ‘60s^3^, optical fibres have attracted much interest for their integration in challenging environments.

Among the Optical Fibre Sensor (OFS) techniques, those based on scattering phenomenon in pure or doped amorphous silica have been shown to successfully monitor environmental parameters such as temperature, strain, pressure, etc^4^. Optical fibre properties and responses, indeed, depend on temperature and/or strain and therefore the fibre itself can be used as the sensitive element of the sensor. Different classes of fibre-based sensing techniques have recently been investigated under extreme environments such as Fibre Bragg Gratings (FBGs) for discrete and spatially localised measurements^5–7^ whereas Brillouin^8, 9^, Raman^10, 11^ and Rayleigh^12, 13^ scattering phenomena are exploited to design distributed sensors of various environmental parameters such as temperature and/or strain. While Brillouin and Raman sensor spatial resolutions remain in the range of one meter, the advantage of Optical Frequency Domain Reflectometry (OFDR) is that it offers the best spatial resolution of a few µm over 70 m of fibre length^14, 15^. Furthermore, Brillouin based sensors are affected by radiation that induces an additional Brillouin frequency shift (BFS) leading to a direct error in the temperature and strain measurements^8, 9^. Differential Radiation Induced Attenuation (RIA) between the Stokes and Anti-Stokes signals leads to a strong degradation of single-ended Raman Distributed Temperature Sensors (RDTS)^10, 11^ thus inducing large errors in the measured temperature. On the other hand, recent work from our group has shown that radiations at MGy dose levels do not affect the optical fibre Rayleigh signature at the basis of the OFDR technique. Indeed, temperature and strain coefficients remain unchanged, within the 5% error, up to 10 MGy for a large variety of standard fibre classes^16^ and distributed temperature measurements (from −40 °C up to 250 °C) have been shown not to be influenced by radiations up to 1 MGy^17, 18^. However, it has been shown that the potentialities of OFDR sensors are affected by Radiation Induced Attenuation (RIA) phenomenon^16^, which limits the sensing range of the fibre, while its packaging could modify the sensor calibration curves and consequently influences the distributed measurements^18, 19^.

In this paper we tested and validated, for the first time to our knowledge, a radiation resistant OFS prototype able to measure both the water level and the temperature inside the SFPs of a NPP. To be implemented this environment such a sensor have to possess a good spatial resolution for the water level measurement (of the order of the cm) and it has to be able to withstand radiation doses in the MGy(SiO_2_) range. Different techniques have been proposed in literature for the liquid level measurements such as Surface Plasmon Resonance (SPR)^20^ and Optical Time Domain Reflectometry (OTDR)^21^ optical fibre based sensors. SPR sensors, which achieve millimetre resolution, are difficult to implement inside a NPP and literature does not state about their radiation hardness. Concerning the OTDR sensors, their spatial resolution being 20 cm for the best ones, they are not suitable candidates for high spatial resolution measurements. Moreover with both SPR and OTDR sensors the simultaneous temperature and water level measurement with one sensing fibre is not allowed, thus making OFDR sensors the best candidates for SFP environments.

**Figure 1:**
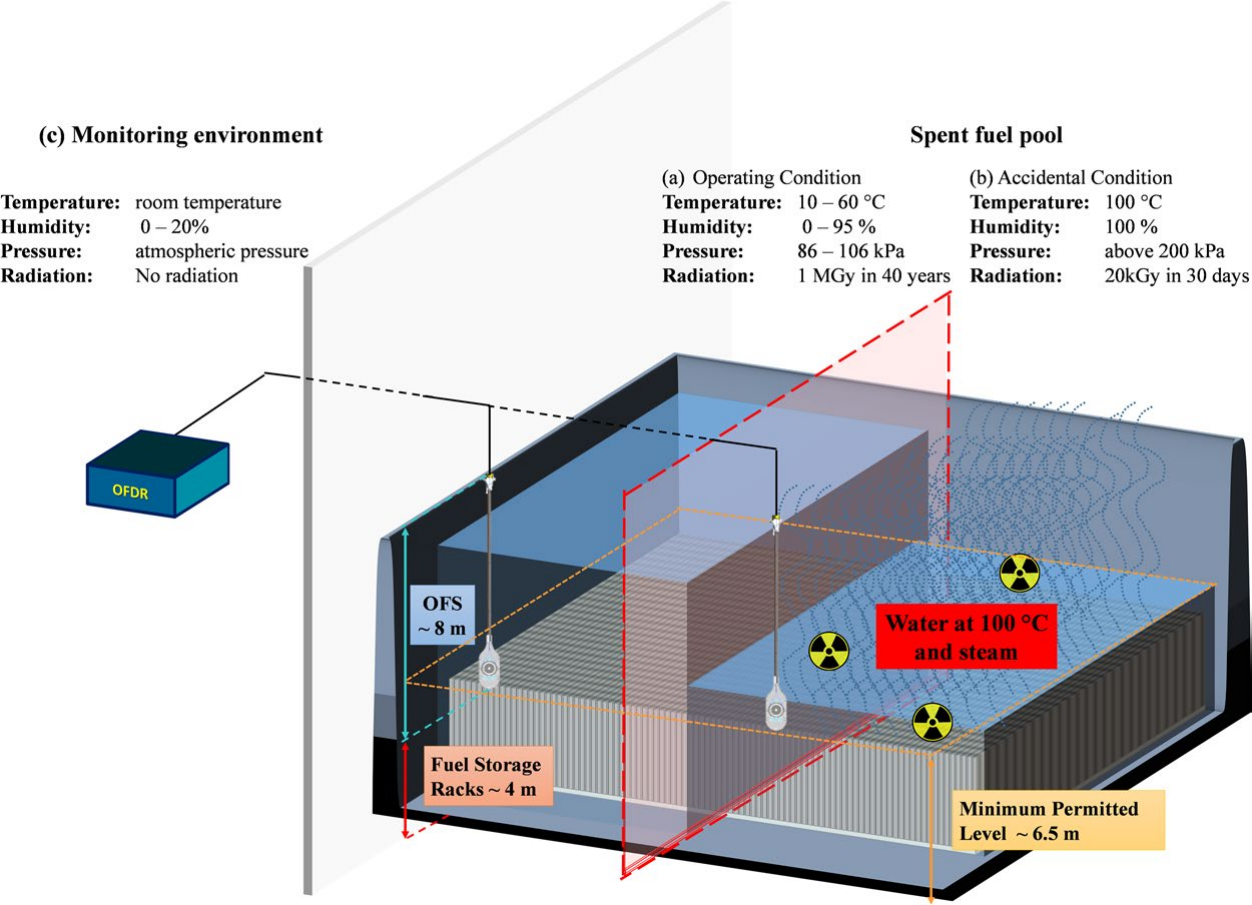
Schematic representation of a SPF: (**a**) the operating condition, characterized by a water temperature from 10 to 60 °C, pressure from 86 to 106 kPa and radiation dose rate of 3 Gy/h leading to a total accumulated dose of about 1 MGy (SiO_2_) for 40 years^23, 24^. In these conditions the evaporation of the water is negligible. (**b**) The accidental condition presents water temperatures up to 100 °C with the presence of boiling water and steam which can lead to an important reduction of the water level inside the pool. The radiation dose rate is ~0.03 kGy/h which means a total accumulated dose of ~20 kGy (SiO_2_) after only 30 days^1, 2^.

In Fig. 1 the schematic representation of such a SFP is reported having a typical depth of 12 m. The storage racks, containing the used fuel assemblies, are placed at the bottom. The fuel assemblies, removed from the reactor after two years of operation, are stored underwater for several years before being sent for reprocessing in a dedicated nuclear facility. The role of water is to both cool the fuel and provide a biological shielding from radiations. As gamma photons mean free path in water barely exceed 50 cm^22^, few meters of water are sufficient for an efficient protection. In nuclear plants, the water level in the SFP is chosen so that the depth of water between the fuel assemblies and the surface remains higher than 2.5 m during handling operations. As fuel assemblies are raised from their full height during their manipulation (4 m), the water level above the fuel assemblies at rest must be at least of 6.5 meters. This ensures that radiations are kept below an acceptable level with regards to radioprotection policy, in any operational conditions, without any specific additional shielding^23, 24^.

In this configuration i.e. in operating conditions (Fig. 1(a)) the OFS has to resist to temperature up to 60 °C and humidity conditions up to 95%, pressure up to ~100 kPa and total accumulated dose of about 1 MGy after the entire operating period of about 40 years^24^ whereas the OFDR interrogation unit can be located in an adjacent building in an instrumentation zone without these severe environmental constraint. In case an accident occurs, the environmental parameters will suddenly change. A possible scenario following a loss of all power supplies is the lack of the water cooling inside the SFP, which causes the water temperature to increase up to 100 °C after 3 or 4 hours. In these conditions the water evaporates continuously and, if the loss of power continues (i.e. no cooling water), its level decrease can became of major importance for the safety of NPP. Indeed water evaporation occurs when the water temperature is lower than 100 °C, but its impact on water level change is negligible compared to the amount of water in the pool. A continuous evaporation, which happens when the water is boiling, could instead lead to a reduction of the water levels down to the safety threshold that is 2.5 m above the fuel assemblies (see Fig. 1(b))^1, 2^.

Reported experimental results deal with the development of the OFS prototype for such environment, with particular attention to its conception and design. The OFS, tested in representative SFP conditions, excepted radiation environment, is able to withstand also accidental scenarios in response to the nuclear safety needs arising from the Fukushima event: we successfully monitored the temperature and the water level (WL). It is worth noting here that the radiation conditions of a nuclear accident were not present simultaneously with the WL tests, since the radiation constraints associated to a nuclear accident where not reproducible in a laboratory test bench. However, we have coupled WL tests with the evaluation of the radiation response of the optical fibre up to a total dose of 3 MGy (SiO_2_) demonstrating that the developed system can be integrated in NPPs.

## The optical frequency domain reflectometry

The OFDR technique is based on the frequency modulated continuous wave technology and involves Fourier transform of interference fringes from the fibre under test and a reference arm. The feasibility of distributed strain measurements with OFDR was published by Froggat and Moore^25^; the authors deduced that the intensity of the backscattered light as a function of wave number is related to permittivity variation along the fibre length. Moreover, since the spatial distribution of the reflection pattern contains both amplitude and phase information for every measurement, they explained that the perturbation caused by an applied strain results in a Rayleigh backscattering spectral shift that can be measured by taking the cross-correlation between the unperturbed and perturbed traces.

**Figure 2:**
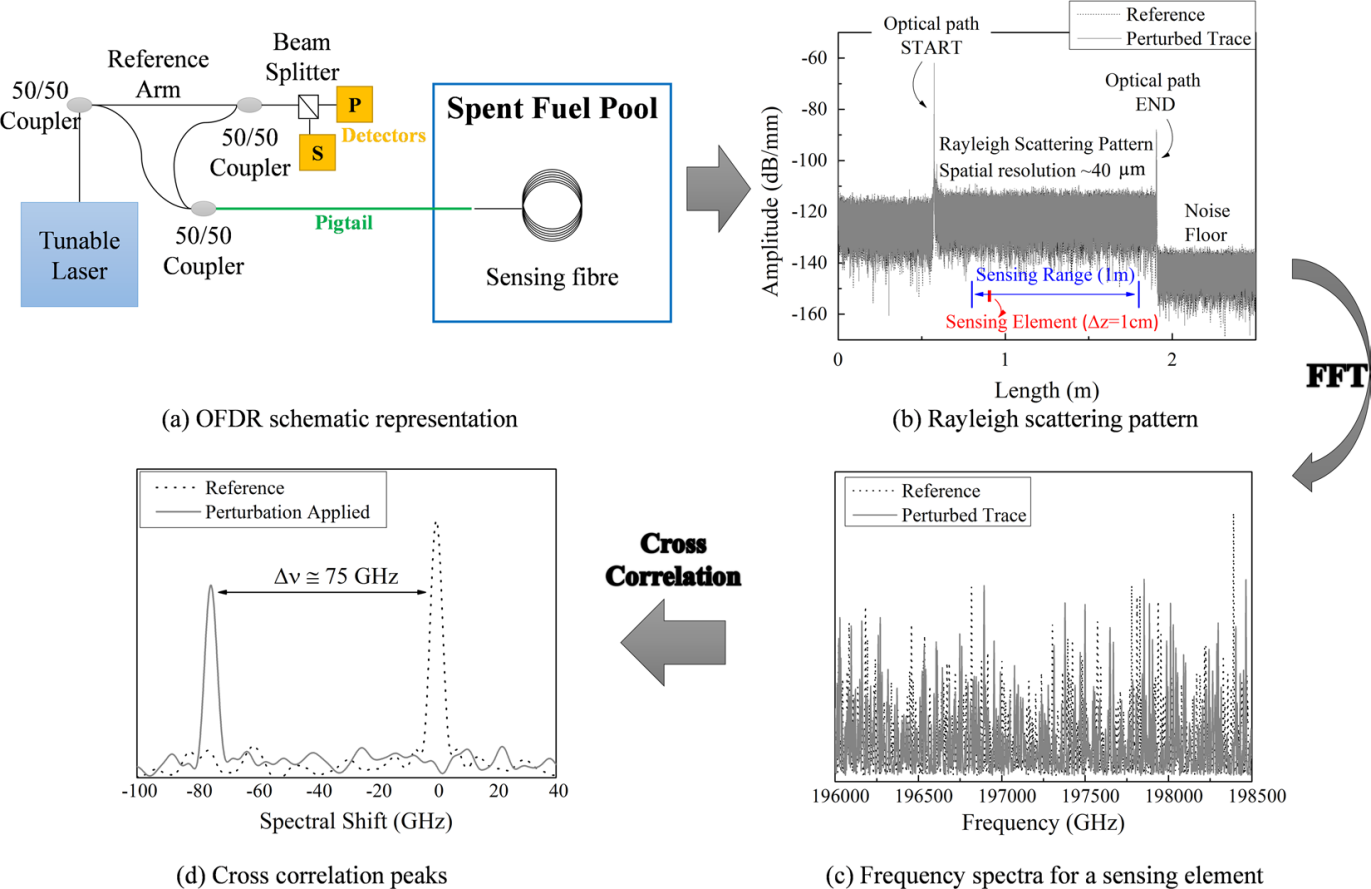
OFDR measure: from Rayleigh pattern to spectral shift information. (**a**) Schematic representation of OFDR system interrogating the sensing fibre which Rayleigh recorded pattern is reported in (**b**) for a reference state (dotted black line) and perturbed state (red line). The information carried in the phase is reconstructed, for each sensing element, by a Fourier transform. (**c**) Frequency spectra for one sensing element. (**d**) Cross-correlation of frequency spectra with respect to the reference one.

The operation of OFDR system is based on finding a beat frequency of the interference fringes formed by mixing together a signal wave coming from the sensing fibre and a reference one^26^. In Fig. 2(a) we reported the schematic representation of the OFDR system. The linearly modulated frequency of light launched by the tunable laser source into the fibre is split in the two arms of the interferometer and then recombined by two 50/50 couplers. The resulted interference fringe is recorded at the detectors in the two polarization states.

The beat frequency of the interference fringes is related to the phase difference between the transmitted signal and the reference waves, obtained by the linear sweep of the laser source into the two arms and containing the reflector positions information^26^. The signal wave is scattered from the whole fibre and a fraction of the scattered light is guided in the backward direction. Being *z* the distance along the fibre length, the time needed by the light to cover twice the distance (in forward and backward directions) is:

1 $$\,\tau =\frac{2{n}_{g}z}{c}$$

where ${n}_{g}$ is the group refractive index of the medium and $c$ is the speed of the light.

At the interferometer the backscattered signal is mixed with the reference signal giving a complex interference fringes. Then, we can write the intensity at the coupler mixing together the electrical field of the reference, *E*_*R*_, and the fibre, *E*_*S*_, arms:

2 $$I(t)={|{E}_{R}(t)+{E}_{S}(t,\tau )|}^{2}$$

Equation (2) represents the interference fringe detected by OFDR and it consists of a continuum of frequencies within the range from zero (that corresponds to the beginning of the optical fibre) to the beat frequencies corresponding to the end of the tested sample. Since the beat frequencies are linearly proportional to the distance at which the light is scattered, the beat frequency distribution also represents the spatial distribution of backscattered light that can be calculated from the Fourier transform of (2)^27^. The resulted Rayleigh scattered pattern is shown in Fig. 2(b) for a 2 m long sample.

Because of the random nature of the reflected spectra, to obtain the information of the perturbation applied on the fibre the two stored traces (reference and perturbed state) have to be correlated^25^. In the scatter profiles as a function of the optical fibre length (Fig. 2(b)) it is chosen the sensing range in which the perturbation is applied. This sensing range, from the two data sets, is then compared in increments of ${\rm{\Delta }}z$, which represent an individual sensing element^25^. For each segment, the inverse Fourier transform is performed to obtain the complex data set in the frequency domain, reported in Fig. 2(c) for both reference and perturbed trace.

When a segment of fibre experiences an external perturbation (for example it is heated or strained), its refractive index or its physical length is modified thus changing locally the optical path of the reflected light traducing in an optical frequency shift. The cross-correlation of the complex data obtained from the inverse Fourier Transform is performed between reference state and the perturbed one: the obtained correlation peak for the two traces is reported in Fig. 2(d) and result in a spectral shift in agreement with:

3 $$\frac{{\rm{\Delta }}\lambda }{{\lambda }_{C}}=-\frac{{\rm{\Delta }}\nu }{{\nu }_{C}}={C}_{T}{\rm{\Delta }}T+{C}_{\varepsilon }\varepsilon $$

where *λ*_*C*_ and *ν*_*C*_ are the mean optical wavelength and frequency, and *C*_*T*_ (*C*_*ε*_) are the temperature (strain) calibration coefficients, respectively. These coefficients are somewhat dependent on the dopant species and concentrations in the core of the fibre, but also on the composition of cladding and coating. The characteristic values for most germanosilicate core fibres are *0.780 µɛ* ^−*1*^ for *C*_*ε*_ $,$ and *6.45∙10* ^−*6*^ *°C* ^−*1*^ for *C*_*T*_^14, 15^. To make a distributed measurement, then, one simply measures the shift by the cross-correlation peaks for each sensing element along the chosen sensing range.

We note that the shift in temperature or strain is purely a linear scaling of the spectral frequency shift Δν for moderate temperature and strain ranges. This dependence, linked to the material properties, has been experimentally proven^14–19^ and the temperature calibration reported in the experimental section (see the first figure of Distributed temperature measurements section) is a clear demonstration of it. Indeed, as explained above the spectral shift is dependent on physical length changes. Looking to the temperature, for range up to 250 °C^18^, the thermal dilatation induces a change in the fibre length proportional to the temperature difference. In the same way for strain range up to 5000 µε^14–16^, since the strain is equal to ε = ∆L/L where L is the fibre length, the frequency shift is proportional to the physical length changes.

## Results and Discussion

### Radiation Induced Effects

For harsh environment applications, the long distance probing fibre length is a key aspect to investigate as OFSs are affected by the RIA that limits their sensing length and degrades the signal-to-noise ratio. Radiation, indeed, induces point defects that affect and move down the optical fibre response which depend i.e. on the nature of the irradiation, the total accumulated dose, the dose rate, the optical fibre core/cladding composition^28^ and of course on the spectral domain of interest. Moreover, irradiation temperature is also a crucial parameter for optical fibres performances: *Girard et al*.^29^ show that there is a complex combined influence between this parameter and the radiation (dose, dose rate). It is, indeed, mandatory to investigate on radiation resistance of tested optical fibre to evaluate its radiation performances.

**Figure 3:**
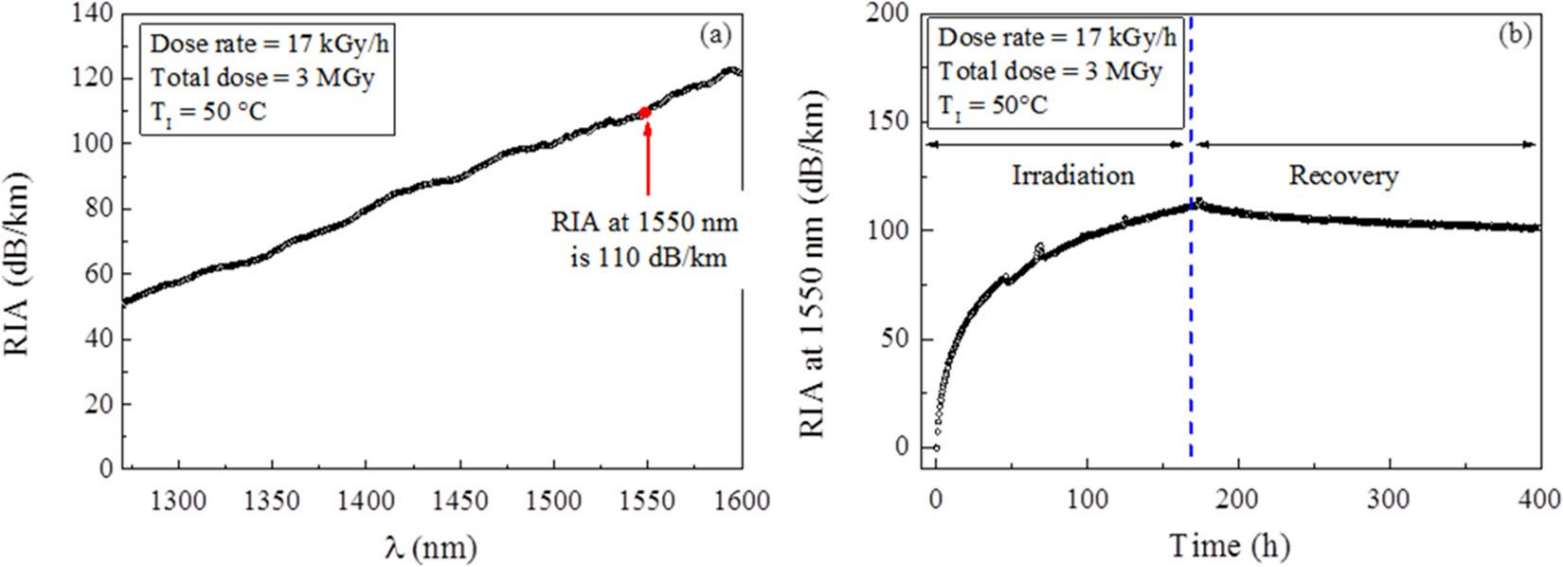
Radiation Induced Attenuation evaluation. (**a**) IR spectral RIA after γ-irradiation at 3 MGy (the dose-rate is 17 kGy/h and the irradiation temperature is 50 °C) ad (**b**) RIA evolution at 1550 nm as a function of the time during irradiation up to 3 MGy and recovery.

We performed *in situ* RIA measurements on the sample to better understand its influence in our sensors. Figure 3(a) reports its spectral evolution after 3 MGy γ-irradiation dose with a dose rate of 17 kGy/h, whereas in Fig. 3(b) the time kinetic of RIA at 1550 nm is shown. The results highlight that at the end of irradiation RIA at 1550 nm is ~110 dB/km and that this value slightly decreases during the recovery thus stabilizing at ~ 100 dB/km at the end of the observation (i.e. after 400 hours). We note also that some ripples are present in the kinetic probably due to environmental changes occurred either at the light source level or at the switch levels.

It is worth noting that the performed tests are done with a much higher dose-rate than those encountered during the actual service in SFPs (i.e. ~3 Gy/h during operating conditions and ~0.03 kGy/h during accidental ones) to perform RIA testing in a reasonable time thus allowing to overestimate the optical losses with respect to the real application^30^. Nevertheless, obtained values allow using tested optical fibre for our purposes. Indeed, considering a maximum device length of 70 m (which preserves the OFDR spatial resolution measurements of 40 µm) and its optical budget (i.e. ~10 dB), the maximum permitted losses along the fibre length are 140 dB/km thus allowing the use up to 90 m of fibre as sensing element for water level and temperature sensors in SFPs.

As already mentioned in the introduction, the packaging of the sensor could modify its calibration curves and consequently can influence the distributed measurements. At the first order, this modification occurs in the fibre coating which could be modified if irradiated^19^. Nevertheless, radiation does not affect the calibration coefficients of a polyimide coated-fibre (i.e. the one studied in this work) since the C_T_ coefficients remain unvaried within 1% fluctuations up to 10 MGy^31^ ensuring that the developed OFS is not submitted to any radiation influence derived both from fibre composition and fibre coating.

### Sensor Prototype design

**Figure 4:**
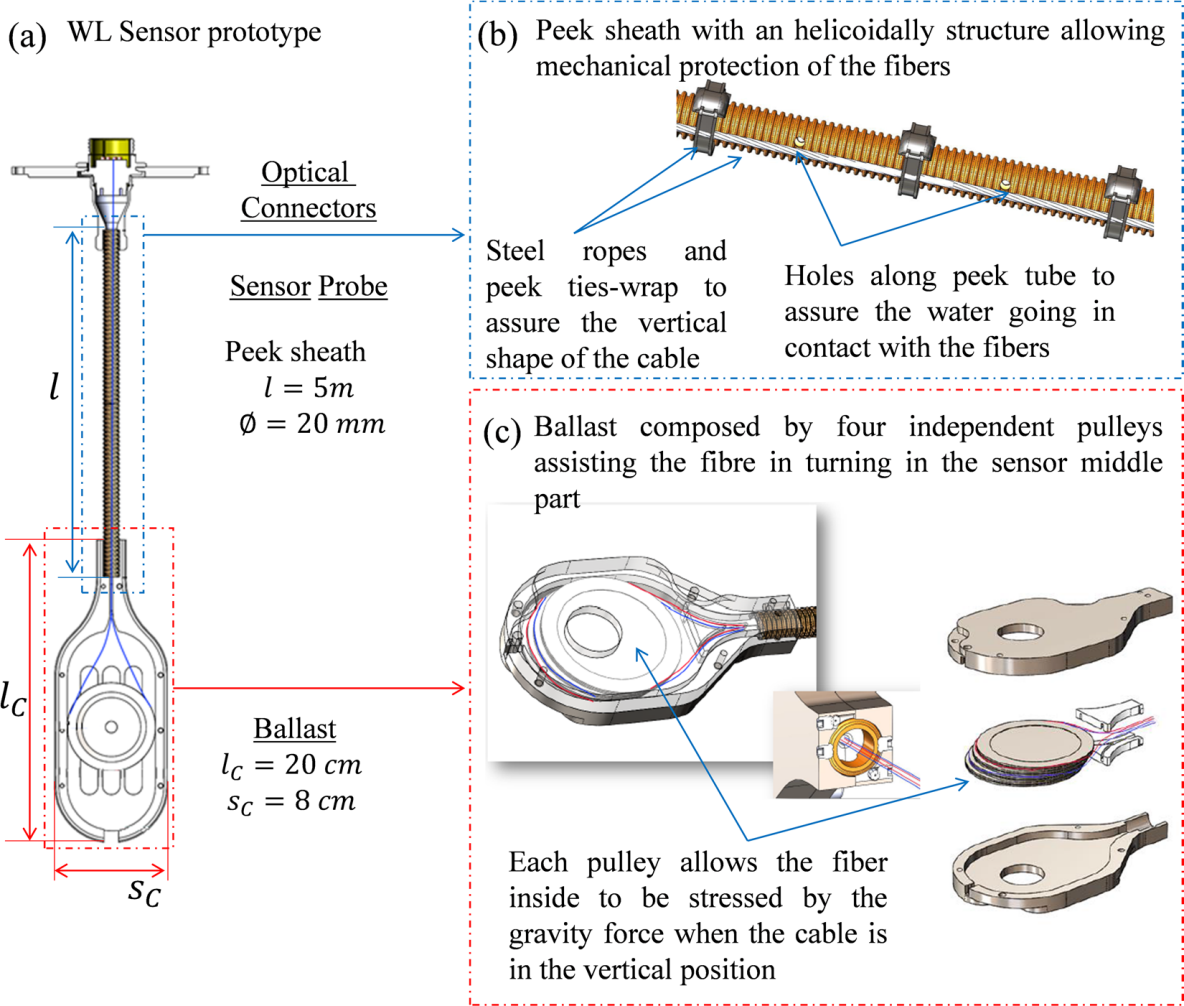
WL sensor prototype technical layout, (**a**) where the three principal parts are highlighted, i.e. the optical connector subassembly, the sensor probe cable and the ballast. The fibre path is represented by the blue line inside the cable. (**b**,**c**) Characteristics of the central cable and the ballast.

The OFS prototype presented in this study was conceived for a dedicated test bench allowing the simulation of a SFP environment (temperature, pressure, humidity…). The OFS cable design for the prototype is shown in Fig. 4. It consists of a dedicated cable (see Fig. 4(a)), which can contain up to four optical fibres, structured in three main parts: the optical connector, the central cable (i.e. the measurement probe, detailed in Fig. 4(b)) and the ballast (Fig. 4(c)).

The top extremity of the cable is the optical junction between the sensor probe and the OFDR setup, located in the instrumental zone (with limited harsh constraints) with respect to the test bench place. To connect OFDR instrument to the sensor probe, we used a 20 m long optical harness including four optical fibres.

The sensor probe is made in PEEK (a colourless organic thermoplastic polymer chosen for its nominal resistance to radiation and temperature) with a helicoidally structure that allows more flexibility of the cable. It is 5 m long with a diameter of 2 cm thus allowing the fibres to be used as in the bare conditions. This is very important to ensure that no local strain is applied from the cable to the sensing fibres when it dilates, possibly causing measurement inaccuracies. The presence of the PEEK protection sheath can, however, influence the temperature response of the sensing fibre that is not directly in contact with the external environment (water). To overcome this problem we added small holes along the tube length to ensure the penetration of the water inside the sheath, in contact with the sensing fibre (see Fig. 4(b)). This design is also useful in accidental conditions since the amplitude of water movements induced by the boiling water will be limited inside the tube of a limited diameter. Figure 4(b) also shows another characteristic of the cable, essential for a fine measure of the liquid level: it is indeed kept vertically thanks to two steel ropes and PEEK tie-wraps. Such a condition is crucial for an accurate measure of the temperature profiles and ensures that the fibres are not in physical contact with the protecting PEEK sheath and that they remain in a well determined strain configuration.

The ballast, detailed in Fig. 4(c), is positioned at the end of the PEEK cable. Internally, it is composed by four independent pulleys each one hosting one fibre. The pulleys have then dual function: first they assist the fibre in turning in the sensor middle part; for this reason it is needed that they are independent from each other and from the ballast structure. Indeed, each of the four fibres inside the sheath needs to be free from the others. Second, the pulley allows the hosted fibre to be stressed by the gravity force when the cable is in the vertical position. The ballast dimensions are chosen in order to avoid transmission losses due to the fibre bending, which can occur if, for example the pulleys diameter is too short. For this, the ballast width, s_C_ in Fig. 4(a), is chosen ≥ 5 cm which is the minimum possible diameter allowed to avoid bending losses in the fibre path. In the tested prototype s_C_ = 8 cm and, in order to be able to maintain the prototype in the vertical position during the test, even in accidental conditions, the length of the ballast, l_C_ is 20 cm.

It is worth noting that, for the integration of such a sensor in NPPs, its length as well as the ballast dimension, has to be adapted to the needs of the real environment. In particular, since the pool is 12 m deep and the fuel racks cover the first 4 m (from the bottom of the pool), a sensor length of 8 m is needed. This will be traduced in a sensor probe of 8 m which hosts a total fibre length of ~16 m (probe + ballast).

### Distributed temperature measurements

To determine the temperature profile along the prototype sensor length, a calibration procedure was developed. Ten thermocouples are placed along the test bench wall (but not inside the OFS cable). Their temperature measure serves as reference to verify the OFS data and for the sensor calibration (i.e. to determine the temperature coefficient (C_T_) of the fibre cable from spectral shift responses following Eq. (3)). Indeed, to our knowledge, this is the first time that such an OFS has been tested in real conditions and in its almost final version.

**Figure 5:**
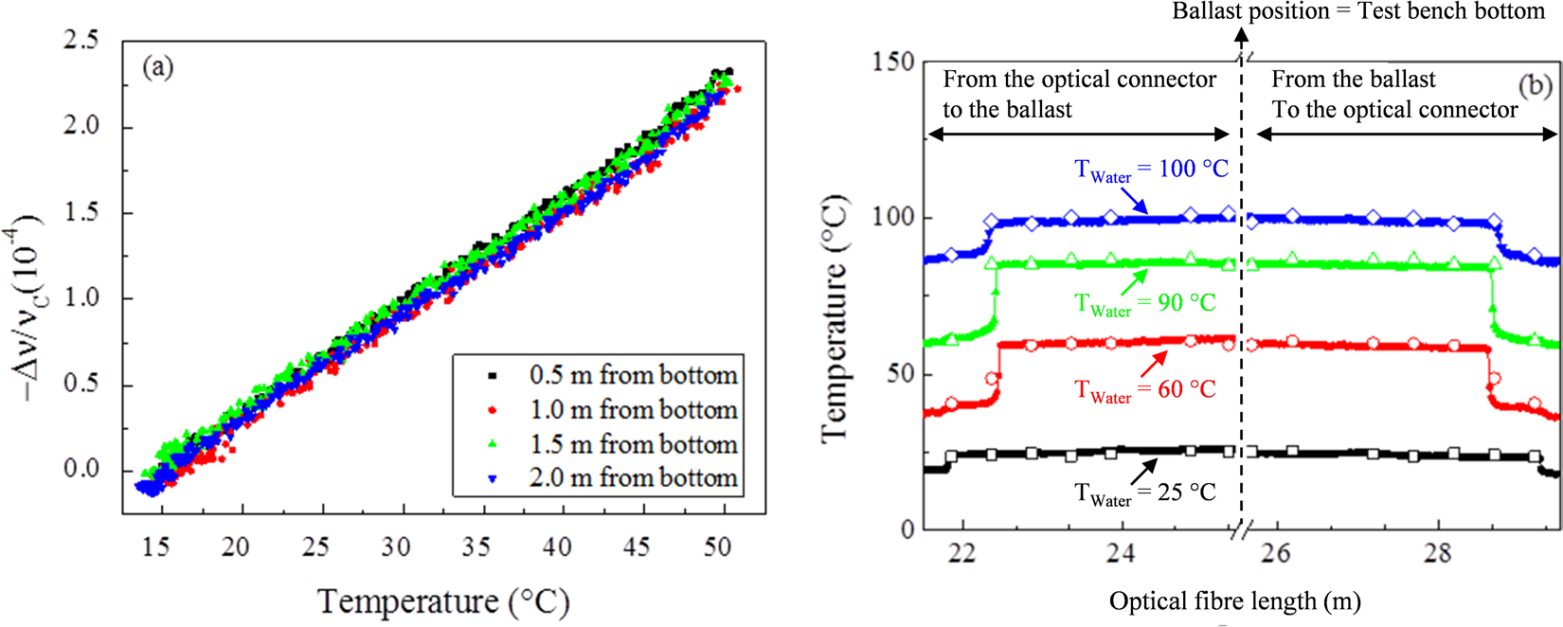
Distributed temperatures in operating and accidental conditions. (**a**) Temperature calibration curves during water heating (15–50 °C) around four different thermocouple positions along the test bench height. (**b**) Temperature evolution along the fibre length inside the test bench (sensor probe) at four different situations during the experiment: operating condition with water at ~25 °C (black solid dots), operating condition with water at ~60 °C (red solid dots), accidental condition with water at ~90 °C and (green solid dots), accidental condition with water at ~100 °C, presence of steam inside the chamber and boiling water (blue solid dots). The open dots in each curve represent the temperature values detected with the thermocouples placed along the test bench height.

Since the strain was maintained fixed along the sample, the spectral shift was substantially induced by the temperature variation of the surrounding water. Figure 5(a) reports the corresponding calibration curves for four different positions along the sensor length and near the thermocouple. The obtained C_T_ value is (*6.36* ± *0.02*)*∙10* ^−*6*^ *°C* ^−*1*^. Distributed temperature profiles as a function of the optical fibre length are displayed in Fig. 5(b). We note that the connection to the OFDR device is done via a 20 m long optical harness so that the sensing fibre starts at ~ 20 m (i.e. the location of the optical connectors). Then, we have 10 meters of sensing fibre: 5 m from optical connectors to the ballast and another 5 m from the ballast to the optical connectors. The prototype was indeed designed to have a roundtrip thus allowing a double measurement of the temperature profile between 20 m and 30 m. Temperature values recorded by the thermocouples (empty dots on each curve) are displayed in Fig. 5(b) in order to have a direct comparison of the experimental data obtained via the OFS. Four different conditions are represented: the black and the red symbols correspond to the nominal operating conditions at two different temperatures, ~25 °C and ~60 °C respectively. Green and blue curves are referred to accidental conditions: the first during a temperature increase (~90 °C, with negligible evaporation), the second in presence of boiling water and steam. The data of spectral shift obtained at the ballast level (i.e. the extremity of the OFS) are removed from Fig. 5b) (see the cut in the middle of the graph). Indeed, in this part of the sensing fibre, the effect of the bend around the pulley misrepresents the temperature information and it is not useful for the estimation of the temperature along the fibre.

The reported results highlight that the distributed measurements of the sensor prototype agree well with the local ones of the thermocouples in almost all the conditions. The temperature differences between the sensor prototype and thermocouples are below 1 °C for the part inside the water, whereas the differences increase up to 2 °C in the zone outside the water for all the conditions. The accuracy of the measurements, calculated by the statistical analysis of the dispersion of the temperature measurements (i.e. taking the standard deviation of the temperature measurements along the fibre length), is about 0.2 °C under both operating and accidental conditions (when the water is not boiling); it reaches 0.5 °C in presence of boiling water, since in this case the signal-to-noise ratio of the traces increases due to the movement of the water inside the test bench.

### Water level results

**Figure 6:**
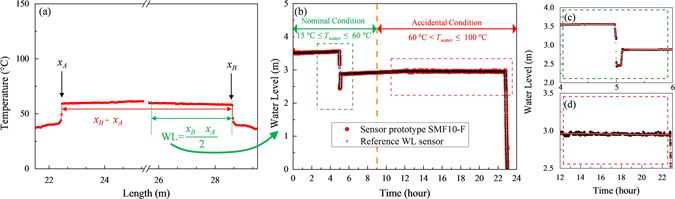
Water level measurement: (**a**) Temperature profile for one of the registered measurements which shows how the WL is calculated from the temperature discontinuities position *x*_*A*_ and *x*_*B*_. (**b**) Water level as a function of time measured with the sensor prototype (red dots) and with the calibrated reference sensor (black point) during the entire experiment. (**c**,**d**) Are zooms in normal and accidental conditions respectively.

Operatively, WL is directly acquired from the temperature profile along the fibre length, reported for one measurement in Fig. 6(a). From the temperature profile the positions of the temperature discontinuities that appear at the air/water interfaces are calculated (*x*_*A*_ and *x*_*B*_ in Fig. 6(a)). To do so the derivative of the curve represented in Fig. 6(a) is computed and then the position of the local maximum around the temperature gradient of each side is selected for every measurement. Then, the water level is found dividing by two the length between the two discontinuities:

4 $$\text{WL}\,=\,\frac{{x}_{B}-{x}_{A}}{2}$$

Temperature and water level measurement are then dependent but not correlated. We use the distributed temperature measurements along the optical fibre path to locate the water/air interfaces (i.e *x*_*A*_ and *x*_*B*_). These two positions, as the architecture of the OFS is known, give the measure of the water level thanks to Eq. (4).

The increase of the water temperature is accompanied by an increase of the air temperature outside the water. The important point is that, as shown in Fig. 5(b), the temperatures of the water and the air/steam are well distinguishable for each of the conditions (operating and accidental). Indeed, even with the most severe constraint i.e. boiling water, the temperature difference between water and steam decrease is well detectable by the OFDR (~1 °C) being the temperature measurement accuracy of ~0.2–0.5 °C. Consequently, temperature profile measurements can be used to determine the WL within an experimental uncertainty of 1 cm (i.e. the spatial resolution of the distributed temperature measurements and then the uncertainty associated *x*_*A*_ and *x*_*B*_ calculation).

To evaluate the robustness of the prototype, the monitoring of WL was carried out during different runs of 24 hours consisting in diverse phases where the environmental conditions of the sensor prototype changed, simulating the SFP behaviour in operating and accidental scenarios. During the first 9 hours, we controlled WL in normal conditions with water temperature ranging from ~15 °C up to ~60 °C. During this period the water level was manually changed (see the change at around 5 hours) to evaluate the performance of the liquid level sensor prototype. Then, the temperature of the water was continuously increased above 60 °C, representative of an accident in the SFP environment, up to reach the boiling condition. The prototype was tested during one night to check on the validity and the robustness of our OFS as well as to evaluate the stability of the measurements in such conditions. OFS WL measurements were compared to a pressure sensor which measures the amount of liquid inside the bench through the differential pressure between the hydrostatic pressure of the water taken at bottom of the tank and the atmospheric pressure of the air and using Stevin’s law. Indeed, as in the case of temperature measurements, this is the first time that such an OFS has been tested in real conditions and consequently a comparison with a reference sensor type is needed.

The obtained results are reported in Fig. 6 where we follow the evolution of WL with time during the whole run (Fig. 6(b)) and two zooms to better evaluate the performances of the sensor prototype in operating, Fig. 6(c), and in accidental conditions, Fig. 6(d). We see that the monitoring of the water level remains possible during the whole experiment (see Fig. 6(b)), thus demonstrating that the design of the level probe is well adapted to the application. The zooms in both normal and accidental conditions permit to evaluate the sensor prototype performance in terms of OFS time response and of WL measurement accuracy. In normal operating conditions, Fig. 6(c), it is possible to observe the fast response of the OFS (on the order of seconds). The zoom of Fig. 6(d) on the other hand, informs on the robustness of the tested OFS; it is possible to note indeed that the WL measurements are well-established even during the long period when the water was boiling (~10 hours).

The WL accuracy was also evaluated. It is calculated by statistical analysis of the dispersion of the WL measurements both in operating and accidental conditions. By taking the standard deviation of the WL measurements in operating condition (i.e. from 0 to 4 hours and from 5 to 8 hours in Fig. 6(b)) we obtained an accuracy of 1 cm. During accidental conditions (from 10 to 22 hours) we notice that the accuracy reaches 3 cm in presence of boiling water (Fig. 6(c)). It is worth noticing that WL measurements accuracy depends on the environmental conditions and that it increased in accidental condition due to the fact that when the water boils the OFS cable is subjected to the movements of the water in the tank thus increasing the signal-to-noise ratio, which however remains in the cm range. It is worth noting that these results fit with AREVA needs for such a sensor being the WL measurement resolution lower than the one aimed by AREVA (i.e. 6 cm). Indeed, other optical fibre-based liquid level sensors^20, 21, 32, 33^, although possessing more precise sensitivity, are not suitable for NPP applications, since the integration is not easy^20^, their dynamic range is, currently, in the centimetre range^32, 33^ and the spatial resolution is not fine enough for the envisaged application^21^. For all these reason, and noticing that OFDR sensors resist to radiation up to the MGy range^17, 34^, the developed OFSs are then very encouraging for the near-future OFSs integration in NPPs.

## Conclusion

This work has dealt with the conception and validation of an OFS prototype for distributed temperature measurements and WL monitoring inside SFPs. We demonstrated that WL measurements exploiting the OFDR technology can be achieved. Using an optical fibre with controlled strain thanks to an innovative cable structure, we can detect the discontinuities along the fibre temperature profile at the water/steam interfaces and from these measurements determine the water level. To our knowledge such a device is innovative, thus conferring to our study a pioneering character in this field at the cutting edge of the state of the art. With an appropriated test bench, the sensor prototype was tested in both real operating and accidental conditions of a SFP (without taking in account the radiation constraints) highlighting that both distributed temperature and water level measurements are possible with an excellent accuracy up to 0.5 °C for distributed temperature measurements and up to 3 cm for WL. Since this measurement type has to resist to severe constraints associated with high level of radiation, we also investigated the RIA which is the limiting radiation induced effect in relation with these measurements. Its time dependence up to an accumulated dose 3 times higher than the real application, have highlight that radiation does not limit the employment of our OFS. The reported results demonstrate that the proposed prototype device successfully withstands to the harsh environment of SFPs. Therefore, they assume a crucial relevance to ensure the safety in NPPs, positively impacting global the environment safeguards.

## Materials and Methods

Tested sample was a single mode fibre with a low-fluorine (F)-doped core of <10 μm diameter, an F-doped cladding (diameter ~ 125 µm) at higher concentration and a polyimide coating resisting up to 300 °C. No optical attenuation is detected for bending with a radius higher than 2.5 cm.

Distributed temperature and water level measurements were performed thanks to an Optical Backscatter Refectometer (OBR) 4600 from Luna Technologies. In all measurements the laser source was tuned over a spectral range of 21 nm centred around 1550 nm (with an accuracy of 1.5 pm), yielding a nominal spatial resolution of the Rayleigh scatter pattern of 40 µm. The data acquisition rate limits the FUT length to roughly 70 m. Each measurement took less than 5 s for the 21 nm wavelength scan and associated spectral shift calculations.

The test bench used for the measurement is a tube of 5 m long, with a manual system to be entirely filled or emptied; the heating system allows simulating both operating and accidental conditions of a SFP warming the water thanks to 12 resistances disposed at the bottom of the tube. This system permits a very slow increase of the temperature from ~15 °C up to ~100 °C that is reached after 7–8 hours. The water temperature is controlled by 10 thermocouples located every 50 cm along the test bench height thus providing a comparison with the OFDR results.

The γ-ray irradiation was performed using a ^60^Co source facility (BRIGITTE) in SCK-CEN (Mol, Belgium)^35^. The accumulated dose was set to 3 MGy with a dose-rate of 17 kGy/h, whereas the temperature ranges from 30 °C to 50 °C. Time and spectral dependence (700–1700 nm) of the RIA during and after the γ-ray exposure were evaluated with an optical spectrum analyser and a three super luminescent diode sources to cover the entire spectral range.

The reference water level sensor is a Rosemount 3154 N nuclear qualified pressure transmitter used in its differential pressure configuration. The measured pressure range is 500 mbar (with an accuracy of 0.2% at the maximum of the range, i.e. 1 mbar at 500 mbar)^36^. To determine the maximum error on the water level measurement, we used the formula of the relative pressure of a fluid in a tank with atmospheric pressure applied to the free surface:

5 $${\rm{P}}={\rm{\rho }}\,{\rm{g}}\,{\rm{h}}$$

with ρ the fluid density, g the gravity acceleration and h the fluid height. Because of fluid density dependency with temperature the effect of this has to be considered into the reference water level measurements. To do so, the temperature measurements obtained from the thermocouples have been used to take in account this nonlinear dependency of the water density in the level calculation.

## References

[1] National Research Council, Lessons Learned from the Fukushima Nuclear Accident for Improving Safety of U.S. Nuclear Plants, (The National Academies Press, 2014). 25473694

[2] Yukiya, A. The Fukushima Daiichi Accident - Report by the Director General, International Atomic Energy Agency, Vienna (Austria) (2015).

[3] Kao K, Hockham G (1966). Dielectric-fibre surface waveguides for optical frequencies, Proceedings of the Institution of Electrical Engineers.

[4] Bao X, Chen L (2012). Recent Progress in Distributed Fiber Optic Sensors. Sensors.

[5] Morana A (2014). Radiation tolerant fiber Bragg gratings for high temperature monitoring at MGy dose levels. Opt. Letters.

[6] Faustov A (2012). Highly Radiation Sensitive Type IA FBGs for Future Dosimetry Applications. IEEE Trans. Nucl. Sci..

[7] Morana A (2015). Radiation Vulnerability of Fiber Bragg Gratings in Harsh Environments. J. Lightwave Tech..

[8] Phéron X (2012). High γ-ray dose radiation effects on the performances of Brillouin scattering based optical fiber sensors. Opt. Express.

[9] Cangialosi, C. *et al*. Steady state γ-ray radiation effects on Brillouin fiber sensors, *Proc. SPIE*, **9634**, 24th International Conference on Optical Fibre Sensors, (2015).

[10] Cangialosi C (2014). Development of a Temperature Distributed Monitoring System Based on Raman Scattering in Harsh Environment. IEEE Trans. Nucl. Sci.

[11] Cangialosi C (2015). Hydrogen and radiation induced effects on performances of Raman fiber-based temperature sensors. J. Lightwave Tech..

[12] Faustov A (2015). The Use of Optical Frequency Domain Reflectometry in Remote Distributed Measurements of the γ Radiation Dose. Tech. Phys. Lett..

[13] Faustov, A. *et al*. Comparison of simulated and experimental results for distributed radiation-induced absorption measurement using OFDR reflectometry, *Proc. SPIE*, **8794** (2013).

[14] Soller J, Gifford DK, Wolfe MS, Froggatt ME (2005). High resolution optical frequency domain reflectometry for characterization of components and assemblies. Opt. Express.

[15] Soller, J., Wolfe, M., Froggatt, M. E., Polarization resolved measurement of Rayleigh backscatter in fiber-optic components, in OFC Technical Digest, Los Angeles (2005).

[16] Rizzolo S (2015). Vulnerability of OFDR-based distributed sensors to high γ-ray doses. Opt. Express.

[17] Rizzolo S (2015). Radiation Effects on OFDR based sensors. Opt. Letters.

[18] Rizzolo S (2015). Radiation Hardened Optical Frequency Domain Reflectometry Distributed Temperature Fiber-based sensors. IEEE Trans. Nucl. Sci..

[19] Rizzolo S (2016). Radiation Characterization of Optical Frequency Domain Reflectometry Fiber-Based Distributed Sensors. IEEE Trans. Nucl. Sci..

[20] Pozo, A. M., Pérez-Ocón, F., and Rabaza, O. A Continuous Liquid-Level Sensor for Fuel Tanks Based on Surface Plasmon Resonance, *Sensors*, **16**, doi:10.3390/s16050724.C (2016). 10.3390/s16050724 PMC4883415 27213388

[21] Yang S, Chen G (2001). Yang, Fiber optical liquid level sensor under cryogenic environment. Sens. Actuator A-Phys.

[22] NIST: National Institute of Standards and Technology, X-Ray Mass Attenuation Coefficients http://physics.nist.gov/PhysRefData/XrayMassCoef/tab4.html (2009).

[23] U.S.NRC: United States Nuclear Regulatory Commission, Storage of Spent Nuclear Fuel https://www.nrc.gov/waste/spent-fuel-storage.html (*Page Last Reviewed/Updated Wednesday, October 12, 2016*).

[24] Johnson, A.B. Jr. Behavior of Spent Nuclear Fuel in Water Pool Storage, Battelle Pacific Northwest Labs., Richland, Washington (United States) (1977).

[25] Froggat M, Moore J (1998). High resolution strain measurement in optical fiber with Rayleigh scatter. Appl. Opt..

[26] Zheng J (2004). Analysis of optical frequency-modulated continuous-wave interference. Appl. Opt.

[27] Glombitza U, Brinkmeyer E (1993). Coherent frequency-domain reflectometry for characterization of single mode integrated-optical waveguides. J. Lightwave Tech..

[28] Girard S (2013). Radiation Effects on Silica-Based Optical Fibers: Recent Advances and Future Challenges. IEEE Trans. Nucl. Sci..

[29] Girard S (2013). Combined High Dose and Temperature Radiation Effects on Multimode Silica-Based Optical Fibers. IEEE Trans. Nucl. Sci..

[30] Van Uffelen, M. *et al*. Long-term prediction of radiation induced losses in single mode optical fibers exposed to gamma rays using a pragmatic approach, *2000 Radiation Effects Data Workshop 2000*, 80-84 (2000).

[31] Rizzolo S (2016). Investigation of Coating Impact on OFDR Optical remote Fiber-based Sensors Performances for Their Integration in High Temperature and Radiation Environments. J. Lightwave Tech..

[32] Iwamoto K, Kamata I (1992). Liquid-level sensor with optical fibers. Appl. Opt..

[33] Antonio-Lopez JE (2011). Fiber-optic sensor for liquid level measurement. Opt. Letters.

[34] Rizzolo S (2017). Evaluation of Distributed OFDR-Based Sensing Performance in Mixed Neutron/Gamma Radiation Environments. IEEE Trans. on Nucl. Sci..

[35] Fernandez-Fernandez, A. et al. SCKCEN gamma irradiation facilities for radiation tolerance assessment, 2002 NSREC Data Workshop, 171–176 (2002).

[36] Emerson, Rosemount™ 3154N Nuclear Qualified Pressure Transmitter http://www.emerson.com/catalog/en-us/rosemount-3154n-pressure-transmitter (2016).

